# Evaluation of urinary extravasation after non-operative management of traumatic renal injury: a multi-center retrospective study

**DOI:** 10.1007/s00068-021-01825-7

**Published:** 2021-11-22

**Authors:** Arisa Muratsu, Shunichiro Nakao, Jumpei Yoshimura, Takashi Muroya, Junya Shimazaki, Yuko Nakagawa, Hiroshi Ogura, Takeshi Shimazu

**Affiliations:** 1 Department of Traumatology and Acute Critical Medicine, Osaka University Graduate School of Medicine, 2-15 Yamada-oka, Suita, Osaka 565-0871 Japan; 2 Division of Trauma and Surgical Critical Care, Osaka General Medical Center, 3‐1‐56 Bandai‐Higashi, Sumiyoshi‐ku, Osaka, Japan; 3 Department of Emergency and Critical Care Medicine, Kansai Medical University, 2-3-1 Shinmachi, Hirakata, Japan

**Keywords:** AAST grade, Non-operative management, Traumatic renal injury, Urinary extravasation

## Abstract

**Purpose:**

Urinary extravasation is one of the major complications after non-operative management of traumatic renal injury and may lead to urinary tract infection and sepsis. The purpose of this study was to evaluate these factors in patients with traumatic renal injury.

**Methods:**

This was a multi-center, retrospective, observational study performed at three tertiary referral hospitals in Osaka prefecture. We included patients with traumatic renal injury transported to the centers between January 2008 and December 2018. We excluded patients who either died or underwent nephrectomy within 24 h after admission. We investigated the occurrence of urinary extravasation and the related factors after traumatic renal injury using multivariable logistic regression analysis.

**Results:**

In total, 146 patients were eligible for analysis. Their median age was 44 years and 68.5% were male. Their median Injury Severity Score was 17. Renal injuries were graded as American Association for Surgery of Trauma (AAST) grade I in 33 (22.6%), II in 27 (18.5%), III in 38 (26.0%), IV in 28 (19.2%), and V in 20 (13.7%) patients. Urinary extravasation was diagnosed in 26 patients (17.8%) and was statistically significantly associated with AAST grades IV–V (adjusted odds ratio, 33.8 [95% confidence interval 7.12–160], *p* < 0.001).

**Conclusion:**

We observed urinary extravasation in 17.8% of patients with non-operative management of traumatic renal injury and the diagnosed was made in mostly within 7 days after admission. In this study, the patients with AAST grade IV–V injury were associated with having urinary extravasation.

**Supplementary Information:**

The online version contains supplementary material available at 10.1007/s00068-021-01825-7.

## Background

The occurrence of traumatic renal injury has been reported to range between 0.3 and 1.2% of all traumatic injuries, and the most common mechanism is blunt trauma [1, 2]. Traumatic blunt renal injury is treated with operative or non-operative management, with the non-operative management of traumatic blunt renal injury become increasingly apparent [3]. A previous report stated that non-operative management was selected in 96% of all traumatic blunt renal injuries [2].

However, complications associated with non-operative management can occur and require appropriate intervention. A previous multicenter cohort study reported the occurrence of complications to be 32.4% among blunt renal trauma patients [4]. Urinary extravasation is one of the major complications of renal trauma. A previous systematic review reported that the occurrence of the urinary extravasation was 29% among patients with high-grade renal injury [5], and abscess and sepsis may develop if the diagnosis and treatment are delayed [6]. Perinephric infection may lead to tissue fibrosis resulting in urinary obstruction and postrenal nephropathy [7]. One article reported that renal function of the injured kidney with scintigraphy decreased by 10–39% compared with the contralateral kidney [8].

Most of the previous reports on traumatic renal injury focused on risk factors related to the need for nephrectomy [9–11]. However, there is a paucity of the research on urinary extravasation after traumatic renal injury. Thus, the purpose of this study was to evaluate the timing of occurrence and the risk factors relating urinary extravasation in patients with traumatic renal injury.

## Methods

### Study design

This was a multi-center, retrospective, observational study. The study protocol was approved by the institutional review board at Osaka University Hospital (project approval number: 19202-2) and by the institutional review boards of the other participating hospitals. Written informed consent was waived because of the retrospective nature of the study.

### Setting

This study was performed at Osaka University Hospital, Osaka General Medical Center, and Kansai Medical University Hospital. The study period was 11 years from January 1, 2008, to December 31, 2018. These three institutions are tertiary referral hospitals that include an organization specialized in trauma management and intensive care. We diagnosed traumatic renal injury using abdominal computed tomography (CT) scans. Follow-up CT scans were routinely performed about one week after the injury to evaluate the traumatic renal injury and its complications. Follow-up scans were also performed earlier than the routine scans depending on the patient’s clinical condition.

### Participants

We included all patients admitted to the emergency departments of the three hospitals with a discharge diagnosis of traumatic renal injury. We excluded the patients who had either died or undergone nephrectomy within 24 h after admission. We used excretory phase CT scans for the assessment of urinary extravasation if deemed necessary by the evaluating attending physician. Our institutions used 4–5 min excretory-phase scans after contrast material administration. If the urinary extravasation was diagnosed with the earlier phase of scans, excretory phase scans were omitted. Because there was no specific protocol for the timing of follow-up scans about 1 week after the injury, follow-up CT scans were performed based on the decision by the evaluating attending physician.

### Variables

The primary outcome was the occurrence of urinary extravasation, which was diagnosed from abdominal CT scans read by a radiologist and an emergency physician, or by two or more emergency physicians with sufficient reading ability when radiology reports were not available. Timing of occurrence was defined as the time of the first CT scan showing findings of urinary extravasation. We measured the time interval in days from the admission date to the date of the first CT scan diagnosis of urinary extravasation was made. We also examined the factors related to the occurrence of urinary extravasation. The follow-up period was from the date of admission to discharge or the date of the last outpatient visit. The following data were gathered: age, sex, mechanism of injury, medications, systolic blood pressure and heart rate on admission, presence of gross hematuria on admission, blood tests (hemoglobin, blood urea nitrogen, creatinine), severity of the trauma, side of injury, renal injury grade, isolated renal injuries, renal transcatheter arterial embolization (TAE), number of CT scans during hospitalization, length of hospital stay, information on vascular complications, and mortality. The severity of trauma was evaluated based on the Injury Severity Score (ISS) [12, 13]. The grade of renal injury was classified according to the American Association for Surgery of Trauma (AAST) organ injury scales [14].

### Statistical analysis

Continuous variables are presented as the median and interquartile range (IQR) and categorical variables as counts and percentages. We assessed the associations between variables and the occurrence of urinary extravasation using univariate logistic regression analysis and calculated the odds ratio (OR) with 95% confidence interval (CI). We also compared the outcome between AAST I–III injuries with AAST IV–V injuries. In the multivariate analysis, we adjusted the outcome for clinically and statistically significant factors. The interval from admission to the diagnosis of urinary extravasation between two groups was analyzed using the Kaplan–Meier method and the log-rank test. All tests were two-tailed, and *p* values of < 0.05 were considered statistically significant. All statistical analyses were performed with JMP 14 (SAS Institute Inc., Cary, NC, USA).

## Results

**Fig. 1:**
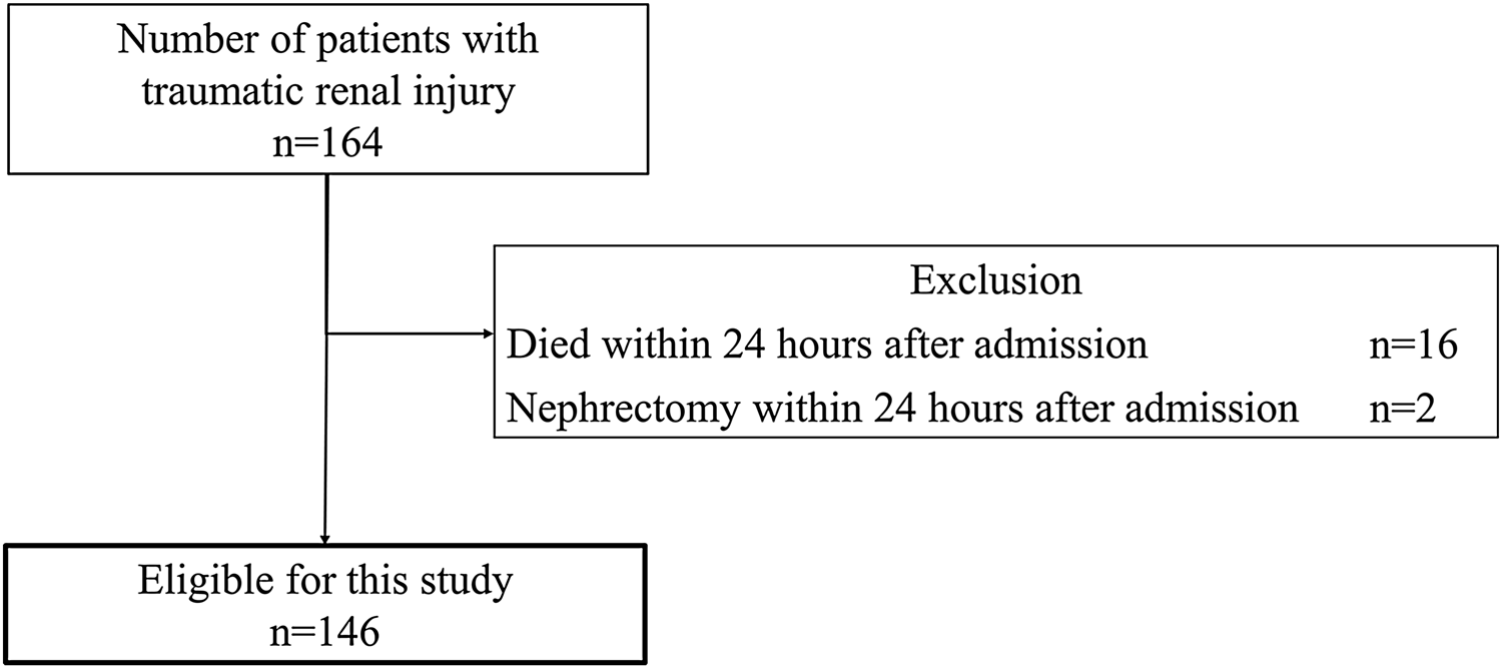
Patient flow diagram

Figure 1 shows the patient flow through our study. During the study period, 164 patients with traumatic renal injury were included. Of them, we excluded 16 patients who died within 24 h after admission and 2 patients who underwent nephrectomy within 24 h after admission. Thus, 146 patients were eligible for analysis.

Table 1 shows the baseline patient characteristics of this study. The median age was 44 (IQR 23–66) years, and 68.5% of the patients were male. The most common mechanism was traffic accident in 65 patients (44.5%), followed by fall on the ground and fall down stairs in 41 patients (28.1%), fall from height in 28 patients (19.2%), and sports-related injury in 4 patients (2.7%). Renal injuries were graded as AAST grade I in 33 (22.6%), II in 27 (18.5%), III in 38 (26.0%), IV in 28 (19.2%), and V in 20 (13.7%) patients. The median ISS on admission was 17 (IQR: 12–29). Renal injuries were left-sided in 87 (59.6%) and isolated in 51 (34.9%) patients. Thirty-two patients (21.9%) required renal TAE on admission, and almost all of them (*n* = 30) had grade III, IV, or V injury (Supplemental Table 1). The median number of CT scans per patient during admission was 3 (IQR 2–4). The median length of hospital stay was 20 days (IQR 11–57), and the mortality rate during hospitalization was 6.2%.

**Table 1:** Baseline characteristics and early complications of the patients with traumatic renal injury

	All patients (*n* = 146)	
Age, years, median (IQR)	44	(23–66)
Male, *n* (%)	100	(68.5)
Mechanism of injury, *n* (%)		
Blunt	142	(97.3)
Traffic accident	65	(44.5)
Fall on the ground or fall down stairs	41	(28.1)
Fall from height	28	(19.2)
Sports-related injury	4	(2.7)
Other	4	(2.7)
Penetrating		
Stabbing	4	(2.7)
Medication, *n* (%)		
Anticoagulant	1	(0.7)
Antiplatelet	9	(6.2)
Gross hematuria on admission, *n* (%)	76	(52.1)
ISS, median (IQR)	17	(12–29)
Injured side, *n* (%)		
Right	60	(41.1)
Left	87	(59.6)
Right and left	1	(0.7)
AAST grade, *n* (%)		
I	33	(22.6)
II	27	(18.5)
III	38	(26.0)
IV	28	(19.2)
V	20	(13.7)
Isolated renal injuries, *n* (%)	51	(34.9)
Renal TAE on admission day, *n* (%)	32	(21.9)
Number of CT scans during admission, median (IQR)	3	(2–4)
Length of hospital stay, days, median (IQR)	20	(11–57)
Mortality, *n* (%)	9	(6.2)
Early complications	30	(20.5)
Urinary extravasation, *n* (%)	26	(17.8)
Diagnosis day, median (IQR)	2	(1–5)
Management, *n* (%)		
Ureteral stent placement	16/26	(61.5)
Nephrostomy	4/26	(15.4)
No procedure	6/26	(23.1)
Vascular complications, *n* (%)	9	(6.2)
Diagnosis day, median (IQR)	7	(1–7)
Diagnosis, *n* (%)		
Pseudoaneurysm	8/9	(88.9)
Arteriovenous fistula	1/9	(11.1)
Management, *n* (%)		
TAE	7/9	(77.8)
No procedure	2/9	(22.2)

*IQR* interquartile range, *ISS* Injury Severity Score, *AAST* American Association for the Surgery of Trauma, *TAE* transcatheter arterial embolization, *CT* computed tomography

Early complications of traumatic renal injury were found in 30 patients (20.5%). The complications included urinary extravasation in 26 patients (17.8%) and vascular complications in 9 patients (6.2%), with both found in 5 patients (3.4%) (Table 1). All the urinary extravasation were found in patients with renal injury of grade III, IV and V. Sixty percent of the patients with grade V renal injuries developed urinary extravasation (Supplemental Table 1).

**Fig. 2:**
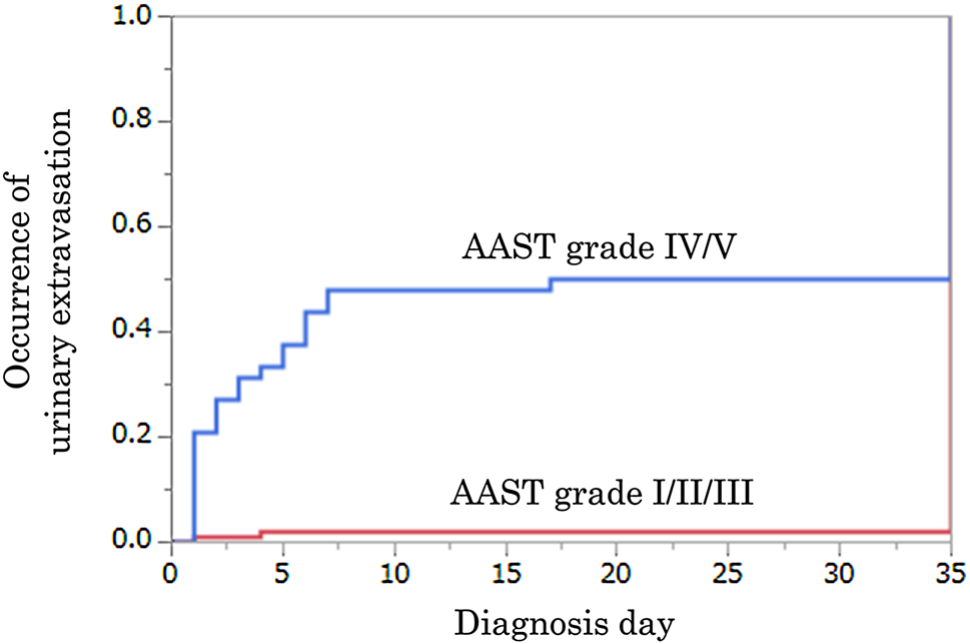
Occurrence of urinary extravasation. Kaplan–Meier curves of time from admission date to the date CT scans showing urinary extravasation were performed. The median number of days to the diagnosis of urinary extravasation in grade IV–V injuries was statistically significantly shorter than that in grade I–III injuries (*p* < 0.001)

The median interval from admission date to the day of diagnosis of urinary extravasation was 2 (IQR 1–5) days, and 25 patients (96.2%) were diagnosed as having urinary extravasation within 7 days (Table 1). One patient with grade V renal injury was diagnosed as having urinary extravasation 17 days after the injury. There was no urinary extravasation found in follow-up CT scans for this patient performed 3 and 6 days after the injury. However, this patient received another CT scan 17 days after the injury since the patient became febrile with leukocytosis and bacteriuria and it showed urinary extravasation. In Kaplan–Meier analysis, the number of days to the diagnosis of urinary extravasation in grade IV–V injuries was statistically significantly shorter than that in grade I–III injuries (*p* < 0.001) (Fig. 2).

Among 26 patients with urinary extravasation, we observed 16 severe urinomas (61.5%) that required stent placement and 4 infected urinomas (15.4%) that required percutaneous drainage. Of them, 15 patients were treated within 24 h from the diagnosis. Vascular complications were found in the patients with grade IV–V injuries. The median interval between admission and diagnosis of vascular complications was 7 (IQR 1–7 days). Of the 9 patients with early vascular complications, 8 had pseudoaneurysms and one had an arteriovenous fistula. Vascular embolization was performed in 7 of these patients (Table 1, Supplemental Table 1).

In a univariate analysis, patients with urinary extravasation showed significant differences in the presence of gross hematuria, renal TAE, AAST renal injury scale, and vascular complications compared with the patients without urinary extravasation (gross hematuria, *p* < 0.001; AAST grade, *p* < 0.001; TAE, *p* = 0.003; vascular complications, *p* = 0.009). There were no significant differences in the blood tests of blood urea nitrogen and creatinine level between the groups. None of the patients with urinary extravasation had deterioration of blood urea nitrogen and creatinine values, and no patients suffered urinary tract obstruction or postrenal nephropathy (Table 2).

**Table 2:** Baseline characteristics and outcomes associated with urinary extravasation

	Urinary extravasation (+) (*n* = 26)		Urinary extravasation (−) (*n* = 120)		*p* value
Age, years, median (IQR)	52	(24–77)	43	(22–65)	0.229
Male, *n* (%)	16	(61.5)	84	(70.0)	0.485
Mechanism of injury, *n* (%)					0.343
Blunt	26	(100.0)	116	(96.7)	
Fall	15	(57.6)	54	(45.0)	
Traffic accident	9	(34.6)	56	(46.7)	
Fall on the ground or fall down stairs	11	(42.3)	30	(25.0)	
Fall from height	4	(15.4)	24	(20.0)	
Sports-related injury	2	(7.7)	2	(6.3)	
Other	0	(0.0)	4	(3.3)	
Penetrating					
Stabbing	0	(0.0)	4	(3.3)	
Medication, *n* (%)					
Anticoagulant	1	(3.9)	0	(0.0)	0.178
Antiplatelet	2	(7.7)	7	(5.8)	0.662
Gross hematuria on admission, *n* (%)	23	(88.5)	53	(44.2)	< 0.001
ISS, median (IQR)	16	(16–22)	19	(10–31)	0.701
Injured side, *n* (%)					0.860
Right	10	(38.5)	49	(40.8)	
Left	16	(61.5)	70	(58.3)	
Right and left	0	(0.0)	1	(0.8)	
AAST grade, *n* (%)					< 0.001
I	0	(0.0)	33	(27.5)	
II	0	(0.0)	27	(22.5)	
III	2	(7.7)	36	(30.0)	
IV	12	(46.2)	16	(13.3)	
V	12	(46.2)	8	(6.7)	
Isolated renal injuries, *n* (%)	17	(65.4)	34	(28.3)	< 0.001
Renal TAE on admission day, *n* (%)	12	(46.2)	20	(16.7)	0.003
Vascular complication, *n* (%)	5	(19.2)	4	(3.3)	0.009
Number of CT scans during admission, median (IQR)	3	(2–4)	2	(1–3)	0.008
Length of hospital stay, days, median (IQR)	25	(16–50)	18	(9–59)	0.290
Mortality, *n* (%)	3	(11.5)	6	(5.0)	0.201

*IQR* interquartile range, *ISS* Injury Severity Score, *AAST* American Association for the Surgery of Trauma, *TAE* transcatheter arterial embolization, *CT* computed tomography

The multivariable analysis adjusted for age, sex, gross hematuria, and AAST grade (grade I–III or grade IV–V) showed that AAST grade IV–V (adjusted OR: 33.8 [95% CI 7.12–160], *p* < 0.001) was statistically significantly associated with the occurrence of urinary extravasation. We observed a trend towards an increased risk of having urinary extravasation in those with gross hematuria (adjusted OR: 3.59 [95% CI 0.860–15.0], *p* = 0.080) (Table3). The complication of urinary extravasation occurred in 56.4% (22/39) of the patients with grade IV–V and gross hematuria.

**Table 3:** Unadjusted and adjusted odds ratios comparing occurrence of urinary extravasation

	Crude OR	(95% CI)	*p* value	Adjusted OR	(95% CI)	*p* value
Age	1.01	(0.99–1.03)	0.229	1.00	(0.98–1.02)	0.836
Sex	0.69	(0.28–1.66)	0.485	1.05	(0.33–3.31)	0.940
Gross hematuria on admission	9.69	(2.76–34.03)	< 0.001	3.59	(0.86–15.01)	0.080
AAST grade IV or V	48.00	(10.60–217.34)	< 0.001	33.80	(7.12–160.59)	< 0.001

*AAST* American Association for the Surgery of Trauma, *OR* odds ratio, *CI* confidence interval

## Discussion

Our study showed that 26 (17.8%) patients had urinary extravasation among 146 patients with traumatic renal injury. We found that patients with AAST grade IV–V renal injury were highly associated with urinary extravasation. Among these 26 patients, 96.2% were diagnosed within 7 days, and urinary stent placement and nephrostomy were performed in 76.9% of them.

The median age of the patients with renal traumatic injury in our study was similar to that of a nationwide study in Japan [15] but was higher than that of a study in the United States [16]. The median ISS and mortality in our study were lower than those of previous studies in Japan and the United States. These differences may have been influenced by hospital characteristics or the exclusion of the patients with nephrectomy or death within 24 h after admission in our study. The occurrence of urinary extravasation was 29% in patients with Grade III–V injury in a meta-analysis and 30.2% in our study, which was quite similar [5].

A previous study showed that urinary extravasation occurred more frequently when there was a rupture of the collecting system and when there was ureteral obstruction [17]. The factors associated with urinary extravasation in our study may be reflecting the depth of renal parenchymal damage because AAST grades are arranged in order of increasing severity according to the depth of injury and the involvement of the urinary collecting system and renal vessels. Grade III is defined as laceration > 1 cm not involving the collecting system, but there are reports that urinary extravasation was found in renal injury of grade III or higher [18, 19]. We found urinary extravasation in two patients among 38 patients with grade III injury. The damage to the collecting system in grade III injury was diagnosed by the presence of urinary extravasation using follow-up CT scans. Therefore, the initial CT scans can miss cases with damages in the collecting system.

In previous studies, urinary extravasation was diagnosed between 3 days and 3 weeks after admission [20, 21]. Most cases of urinary extravasation or vascular complications were diagnosed within 7 days in our study. This may be because we routinely performed follow-up CT scans about one week after the injury or possibly earlier. Additionally, diagnosis of complications may be delayed because of omitted excretory phase scans on the admission day. Although the diagnosis days could be earlier if the excretory phase CT scans were performed in the initial evaluation, excretory phase CT scans may be omitted if the patient condition was not stable or required emergency procedures such as laparotomy. The necessity of follow-up CT scans after renal injury and their timing are still unclear [21, 22]. There are reports that follow-up CT scans were not necessary for patients with hemodynamic stability [20, 21], but there are also reports that a routine follow-up CT examination may be beneficial for the assessment of hematoma expansion in patients with grade IV or V injury [23, 24]. However, there are no reports that describe follow-up CT indications or appropriate timing from the viewpoint of the detection of urinary extravasation. Our study showed that urinary extravasation was observed mostly in patients with grade IV or V injury. Some of these patients required early follow-up CT scans due to concomitant injuries to other organs based on the clinicians’ decisions. Our result may suggest that follow-up CT scans for patients with grade IV or V injury are desirable to evaluate urinary extravasation.

For the management of renal extravasation, a scoping review of adult patients with grade III–V renal injuries reported that urinary stent placement was recommended for cases of persistent fever or abscess formation, which was estimated to occur in 29% of patients with urinary extravasation [5]. Treatment for renal extravasation was performed in 70% (20/26) of the patients with urinary extravasation in our study. To prevent further complications, treatment such as ureteral stent placement was performed at the three centers when urinary extravasation was diagnosed. We did not observe any complications related to the intervention such as urinary infection, renal pelvic or ureteral obstruction, or postrenal nephropathy. We did not observe the deterioration of blood urea nitrogen and creatinine values of the patients and it may be due to the urinary intervention. Our study showed the occurrence and management of urinary extravasation after traumatic renal injury. We also demonstrated the risk factors associated with the occurrence of urinary extravasation. AAST grade IV–V injury and gross hematuria may be useful as indicators for the performance of early follow-up CT scans and early therapeutic intervention for urinary extravasation.

This study has several limitations. First, this was a retrospective observational study with a small number of patients. As the occurrence of renal trauma seemed to be low, a larger prospective study would be needed to confirm our findings. Second, this multicenter study was conducted at only three tertiary care hospitals in an urban area in Japan. Therefore, our inferences may not be generalizable in patients seen at smaller hospitals or in rural areas. Third, we were able to capture patient characteristics and outcomes only from the health records generated during the hospitalization and follow-up visits to outpatient clinics. A more detailed analysis of outcomes will require longer term follow-up of these patients. Fourth, we did not routinely use excretory phase scans on admission day and for follow-up scans and we used 4–5 min excretory-phase scans to diagnose urinary extravasation. Therefore, there may be delayed or missed diagnosis of minor urinary extravasation as the timing of excretory phase CT scans was relatively early in this study. However, clinically important urinary extravasation such as severe urinoma should be captured in our protocol.

## Conclusions

We evaluated the occurrence and the risk factors associated with urinary extravasation after non-operative management of traumatic renal injury. Among the patients with renal traumatic injury, 17.8% of them were diagnosed as having urinary extravasation in mostly within 7 days after admission and 70% underwent urinary intervention without complications. In our study, the patients with AAST grade IV–V injury were highly associated with the occurrence of urinary extravasation.

## Supplementary Information

Below is the link to the electronic supplementary material.

Supplementary file1 (DOCX 29 KB)

## Data Availability

No data and material are available.

## Code availability

JMP 14 (SAS Institute Inc., Cary, NC, USA).
