# Supplementary material for: Evaluation of urinary extravasation after non-operative management of traumatic renal injury: a multi-center retrospective study
Source: Eur J Trauma Emerg Surg. 2021 Nov 22;48(3):2117–24. doi: 10.1007/s00068-021-01825-7 (PMC9192458; doi:10.1007/s00068-021-01825-7)
Supplement: Supplementary file 1 — Supplementary file1 (DOCX 29 KB) [file 68_2021_1825_MOESM1_ESM.docx]

| **SUPPLEMENTAL TABLE 1**. Baseline Characteristics of the Patients with Traumatic Renal Injury by AAST grade | | | | | | | | | | |
| --- | --- | --- | --- | --- | --- | --- | --- | --- | --- | --- |
|  | **I** | | **II** | | **III** | | **IV** | | **V** | |
|  | **(n =33)** | | **(n = 27)** | | **(n = 38)** | | **(n = 28)** | | **(n = 20)** | |
| Age, median (IQR) | 43 | (20-56) | 35 | (18-69) | 45 | (25-64) | 52 | (35-75) | 40 | (24-76) |
| Male, n (%) | 26 | (78.8) | 22 | (81.5) | 23 | (60.5) | 18 | (64.3) | 11 | (55.0) |
| Mechanism of injury, n (%) |  |  |  |  |  |  |  |  |  |  |
| Blunt |  |  |  |  |  |  |  |  |  |  |
| Traffic accident | 13 | (39.4) | 13 | (48.2) | 21 | (55.3) | 11 | (39.3) | 7 | (35.0) |
| Fall on the ground or fall down stairs | 5 | (15.2) | 7 | (25.9) | 12 | (31.6) | 8 | (28.6) | 9 | (45.0) |
| Fall from height | 11 | (33.3) | 3 | (11.1) | 4 | (10.5) | 7 | (25.0) | 3 | (15.0) |
| Sports-related injury | 0 | (0.0) | 1 | (3.7) | 0 | (0.0) | 2 | (7.1) | 1 | (5.0) |
| Other | 4 | (12.1) | 3 | (11.1) | 1 | (2.6) | 0 | (0.0) | 0 | (0.0) |
| Penetrating |  |  |  |  |  |  |  |  |  |  |
| Stabbing | 3 | (9.1) | 1 | (3.7) | 0 | (0.0) | 0 | (0.0) | 0 | (0.0) |
| Vital signs on admission, median (IQR) |  |  |  |  |  |  |  |  |  |  |
| Systolic blood pressure (mmHg) | 123 | (113-145) | 120 | (103-140) | 124 | (106-140) | 139 | (113-162) | 119 | (91-132) |
| Heart rate (/min) | 90 | (77-104) | 100 | (84-115) | 96 | (87-110) | 86 | (68-103) | 79 | (73-96) |
| Medication, n (%) |  |  |  |  |  |  |  |  |  |  |
| Anticoagulant | 0 | (0.0) | 0 | (0.0) | 1 | (2.6) | 0 | (0.0) | 0 | (0.0) |
| Antiplatelet | 0 | (0.0) | 3 | (11.1) | 3 | (7.9) | 2 | (7.1) | 1 | (5.0) |
| Blood test on admission, median (IQR) |  |  |  |  |  |  |  |  |  |  |
| Hemoglobin (g/dl) | 13.5 | (10.9-14.6) | 12.1 | (10.3-13.9) | 11.1 | (9.0-13.1) | 11.3 | (9.9-12.4) | 11.7 | (8.8-13.0) |
| Blood urea nitrogen (mg/dl) | 14.0 | (12.0-16.0) | 16.0 | (13.0-18.0) | 16.5 | (13.0-19.5) | 17.0 | (12.0-20.0) | 15.0 | (13.3-19.0) |
| Creatinine (mg/dl) | 0.74 | (0.63-1.03) | 0.89 | (0.69-0.98) | 0.84 | (0.68-1.18) | 0.81 | (0.66-0.98) | 0.94 | (0.72-1.03) |
| Blood test on day 7 after admission, median (IQR) |  |  |  |  |  |  |  |  |  |  |
| Hemoglobin (g/dl) | 10.1 | (7.9-11.9) | 10 | (8.6-10.7) | 8.7 | (7.9-11.5) | 9.5 | (8.1-10.4) | 9.0 | (8.1-9.9) |
| Blood urea nitrogen (mg/dl) | 13 | (10.5-17.0) | 13 | (11.0-17.0) | 15 | (10.0-19.0) | 13.5 | (10.3-17.0) | 17.0 | (12.3-20.5) |
| Creatinine (mg/dl) | 0.60 | (0.54-0.77) | 0.65 | (0.53-0.76) | 0.76 | (0.57-0.94) | 0.72 | (0.58-1.0) | 0.97 | (0.71-1.05) |
| Gross hematuria on admission, n (%) | 14 | (42.4) | 11 | (40.7) | 12 | (31.6) | 20 | (71.4) | 19 | (95.0) |
| ISS, median (IQR) | 20 | (9-29) | 17 | (9-29) | 18 | (10-30) | 16 | (16-37) | 18 | (16-28) |
| Injured side, n (%) |  |  |  |  |  |  |  |  |  |  |
| Right | 13 | (39.4) | 7 | (25.9) | 19 | (50.0) | 12 | (42.9) | 8 | (40.0) |
| Left | 20 | (60.6) | 20 | (74.1) | 19 | (50.0) | 15 | (53.6) | 12 | (60.0) |
| Right and Left | 0 | (0.0) | 0 | (0.0) | 0 | (0.0) | 1 | (3.6) | 0 | (0.0) |
| Isolated renal injuries, n (%) | 7 | (21.2) | 8 | (29.6) | 13 | (34.2) | 10 | (35.7) | 13 | (65.0) |
| Concomitant injury, n (%) |  |  |  |  |  |  |  |  |  |  |
| Head or neck | 12 | (36.4) | 9 | (33.3) | 8 | (21.1) | 6 | (21.4) | 0 | (0.0) |
| Chest | 13 | (39.4) | 14 | (51.9) | 15 | (39.5) | 12 | (42.9) | 5 | (25.0) |
| Abdomen (except for kidney) | 17 | (51.5) | 14 | (51.9) | 21 | (55.3) | 6 | (21.4) | 6 | (30.0) |
| Liver | 11 | (33.3) | 8 | (29.6) | 15 | (39.5) | 2 | (7.1) | 3 | (15.0) |
| Spleen | 5 | (15.2) | 10 | (37.0) | 10 | (26.3) | 3 | (10.7) | 2 | (10.0) |
| Pancreas | 3 | (9.1) | 0 | (0.0) | 0 | (0.0) | 0 | (0.0) | 0 | (0.0) |
| Intestinal tract | 2 | (6.1) | 0 | (0.0) | 0 | (0.0) | 0 | (0.0) | 1 | (5.0) |
| Bladder or ureter | 1 | (3.0) | 2 | (7.4) | 0 | (0.0) | 1 | (3.6) | 0 | (0.0) |
| Pelvis | 4 | (12.1) | 6 | (22.2) | 6 | (15.8) | 7 | (25.0) | 2 | (10.0) |
| Extremity | 7 | (21.2) | 3 | (11.1) | 6 | (15.8) | 4 | (14.3) | 2 | (10.0) |
| Renal TAE on admission day, n (%) | 2 | (6.1) | 0 | (0.0) | 4 | (10.5) | 11 | (39.3) | 15 | (75.0) |
| Number of CT scans during admission, median (IQR) | 2 | (1-4) | 3 | (2-4) | 3 | (2-4) | 3 | (2-3) | 4 | (3-6) |
| Length of hospital stay, days, median (IQR) | 18 | (8-106) | 18 | (9-50) | 18 | (10-51) | 17 | (11-40) | 39 | (16-68) |
| Mortality, n (%) | 3 | (9.1) | 0 | (0.0) | 2 | (5.3) | 3 | (10.7) | 1 | (5.0) |
| **Early complications** | **0** | **(0.0)** | **0** | **(0.0)** | **2** | **(5.3)** | **15** | **(53.6)** | **13** | **(65.0)** |
| **Urinary extravasation, n (%)** | **0** | **(0.0)** | **0** | **(0.0)** | **2** | **(5.3)** | **12** | **(42.9)** | **12** | **(60.0)** |
| Diagnosis day, median (IQR) |  |  |  |  | 3 | (1-4) | 2 | (1) | 5 | (2-7) |
| Management, n (%) |  |  |  |  |  |  |  |  |  |  |
| Ureteral stent placement |  |  |  |  | 1/2 | (50.0) | 7/12 | (58.3) | 8/12 | (66.7) |
| Nephrostomy |  |  |  |  | 1/2 | (50.0) | 1/12 | (8.3) | 2/12 | (10.0) |
| No procedure |  |  |  |  |  |  | 4/12 | (33.3) | 2/12 | (10.0) |
| **Vascular complications, n (%)** | **0** | **(0.0)** | **0** | **(0.0)** | **0** | **(0.0)** | **5** | **(17.9)** | **4** | **(20.0)** |
| Diagnosis day, median (IQR) |  |  |  |  |  |  | 7 | (4-8) | 4 | (1-15) |
| Diagnosis, n (%) |  |  |  |  |  |  |  |  |  |  |
| Pseudoaneurysm |  |  |  |  |  |  | 5/5 | (100.0) | 3/4 | (75.0) |
| Arteriovenous fistula |  |  |  |  |  |  | 0/5 | (0.0) | 1/4 | (25.0) |
| Management, n (%) |  |  |  |  |  |  |  |  |  |  |
| Embolization |  |  |  |  |  |  | 3/5 | (60.0) | 4/4 | (100.0) |
| No procedure |  |  |  |  |  |  | 2/5 | (40.0) | 0/4 | (0.0) |
| AAST, American Association for the Surgery of Trauma; IQR, interquartile range; ISS, Injury Severity Score; TAE, transcatheter arterial embolization; CT, computed tomography. | | | | | | | | | | |
